# Sedimentary microplastic concentrations from the Romanian Danube River to the Black Sea

**DOI:** 10.1038/s41598-021-81724-4

**Published:** 2021-01-21

**Authors:** Iulian Pojar, Adrian Stănică, Friederike Stock, Christian Kochleus, Michael Schultz, Chris Bradley

**Affiliations:** 1 National Institute of Marine Geology and Geo-Ecology - GeoEcoMar, Str. Dimitrie Onciul 23-25, 024053 Bucharest, Romania; 2 German Federal Institute of Hydrology, Am Mainzer Tor 1, 56068 Koblenz, Germany; 3 School of Geography, Earth and Environmental Sciences, The University of Birmingham, Edgbaston, Birmingham, B15 2TT UK

**Keywords:** Environmental sciences, Ocean sciences

## Abstract

A multitude of recent studies have detailed microplastic concentrations in aquatic and terrestrial environments, although questions remain over their ultimate fate. At present, few studies have detailed microplastic characteristics and abundance along a freshwater–marine interface, and considerable uncertainties remain over the modelled contribution of terrestrial and riverine microplastic to the world’s oceans. In this article, for the first time, we detail sedimentary microplastic concentrations along a River–Sea transect from the lower reaches of a major continental river, the River Danube, through the Danube Delta, the Black Sea coast to the Romanian and Bulgarian inner shelf of the Black Sea. Our results indicate that isolated areas of the Danube Delta are still relatively pristine, with few microplastic particles in some of the sediments sampled.

## Introduction

A multitude of recent studies have detailed microplastic concentrations in aquatic and terrestrial environments, although questions remain over their ultimate fate. At present, few studies have detailed microplastic characteristics and abundance along a freshwater–marine interface, and considerable uncertainties remain over the modelled contribution of terrestrial and riverine microplastic to the world’s oceans. In this article, for the first time, we detail sedimentary microplastic concentrations along a River–Sea transect from the lower reaches of a major continental river, the River Danube, through the Danube Delta, the Black Sea coast to the Romanian and Bulgarian inner shelf of the Black Sea. Our results indicate that isolated areas of the Danube Delta are still relatively pristine, with few microplastic particles in some of the sediments sampled. There are also differences in microplastic composition of beach and marine sediments that likely reflect variations in the original source of the microplastics and local hydrodynamic conditions. Highest microplastic concentrations were found on the Black Sea coast and proximal shelf, indicating the fate of low-density particles in coastal sediments.

Microplastic particles are abundant, ubiquitous and environmentally persistent. A complex contaminant, < 5 mm in size^1,2^, that encompasses a variety of polymers, microplastics are potentially bioavailable and have been associated with a range of adverse biological effects^3–5^. Determining environmental impacts of microplastic particles is complicated due to their diversity of forms (incl. manufactured pellets; micro-beads; fibres; fragments; flakes) and with physical and chemical properties that vary widely according to polymer-type and the presence of additives. Despite a multitude of recent work^6–8^, our understanding of the extent and distribution of microplastics in terrestrial, freshwater and marine environments is inadequate with significant uncertainties over their ultimate fate. More baseline data on microplastic abundance and composition as well as standard protocols for field sampling, laboratory analyses and modelling are needed to ensure comparability between studies, and to aid model development and testing.

While interest in microplastics largely originated from work on marine environments, an increasing amount of studies attests to the importance of terrestrial and freshwater microplastic sources^7,9–11^ with estimated riverine fluxes to the global ocean of 1.15 to 2.41 × 10^6^ t annually^12,13^. These projections are subject to considerable uncertainty (annual riverine plastic flux estimates range between 0.41—4 × 10^6^ t^14^), with respect to microplastic particle transport from source to sink through river catchments, transitional waters, to shallow and deep marine environments. Microplastic abundances are highly variable with concentrations in river sediment ranging from 10 to 100 s of particles per kg^3,15,16^. However, concentrations are higher in urbanised catchment headwaters^6,17^, and in areas of high sedimentation such as freshwater-marine transition zones^8^. These areas constitute potential microplastic ‘hotspots’, which represent a potentially important source of marine plastic.

There is an acknowledged need to link sources and transport pathways, as to date there is little available information on trends in microplastic abundance and composition along the catchment-to-sea continuum. For example, recent surveys of the Rhine and Danube have focussed on aquatic samples^9,18^ which are likely to sample microplastics of low specific density preferentially^19^. Microplastic concentrations are highly variable along individual rivers, reflecting discrete inputs such as effluent from waste water treatment plants (WWTPs)^20^, flow hydraulics and geomorphology, although no relationship between microplastic fibre abundance, WWTPs, or population density was found in the Hudson River, USA^21^. In the Rhine, microplastic concentrations appear to be lower in the Rhine-Meuse Delta^22^ than upstream, but it is unclear whether this can be attributed to reduced (local) microplastic input or to enhanced sedimentation above individual sampling points. The ultimate fate of riverine and marine microplastics is also uncertain given that degradation rates are likely to be highly variable^23,24^ depending upon the molecular weight of individual polymers.

**Figure 1:**
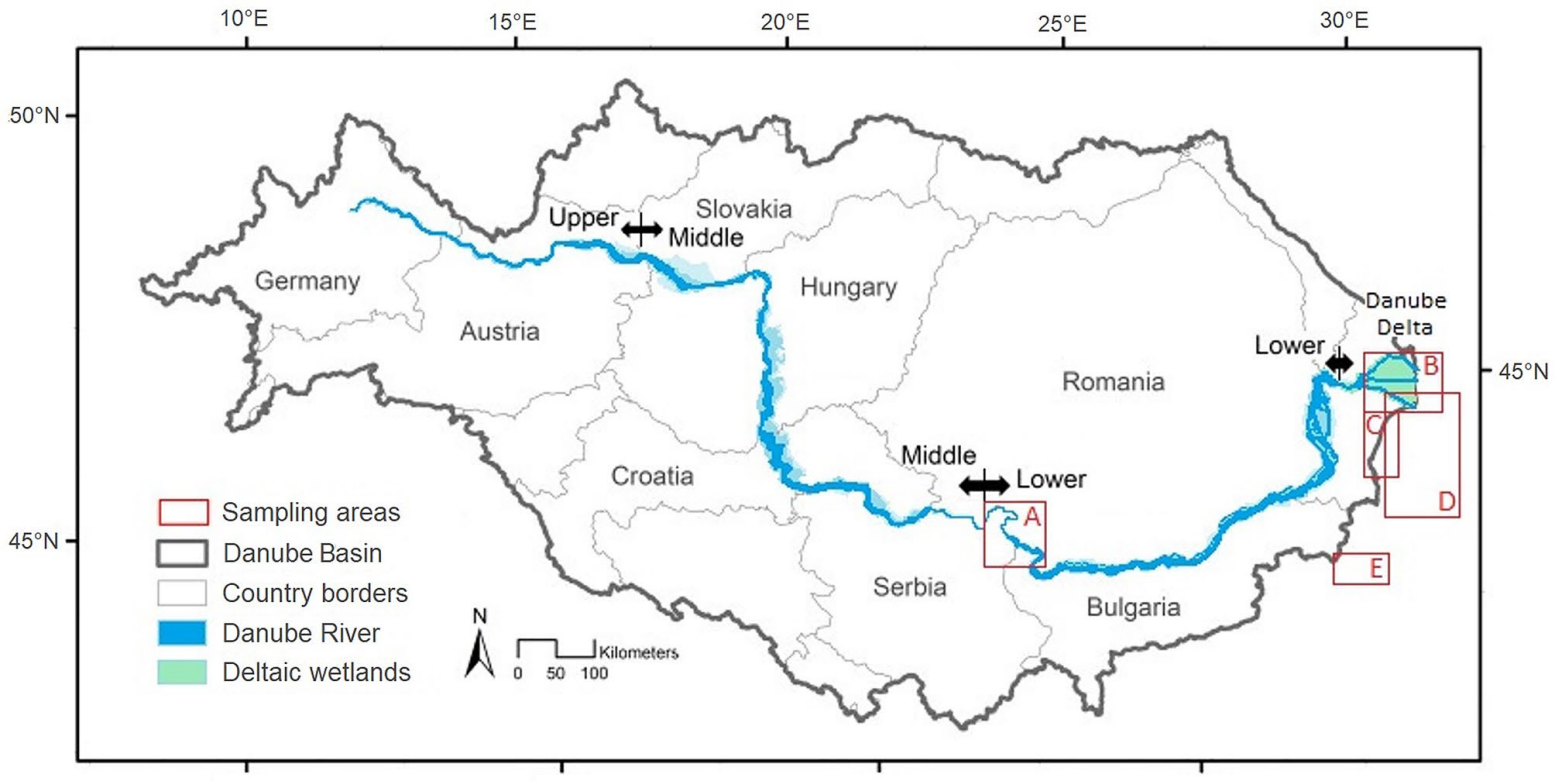
Danube River basin (highlighted) with the marked sampling areas: (**A**)—Iron Gates area, (**B**)—Danube Delta, (**C**)—Black Sea coast, (**D**)—Romanian shelf and (**E**)—Bulgarian shelf (modified from the ref.^47^).

In this article, we provide a first account of microplastic composition and abundance in sediments along a river–sea continuum, from reaches of the Lower River Danube immediately above the Iron Gates I dam, ~ 950 km from the point that the three distributary channels of the Danube discharges into the Black Sea, through riverine and deltaic sites downstream, and along the Romanian and Bulgarian Black Sea coasts to submarine sediments sampled from depths of up to 120 m on the Romanian and Bulgarian Black Sea shelf (Fig. 1). Hitherto, microplastic (and plastic) studies in the catchment have focussed on the Austrian Danube, 1930–1870 km upstream (Vienna to Bratislava), from which estimates of plastic flux to the Black Sea have been derived (1533 t/y)^25^, but the fate of this plastic, and its partitioning between different plastic types and forms is unclear. Accordingly, we sampled sediments from 38 locations comprising: i. marginal river bank deposits along reaches of the Lower River Danube in Romania; ii. distributaries, lakes, lagoons and channel deposits in the Danube Delta; iii. beach deposits along the Danube Delta coast; and iv. continental shelf deposits in the Black Sea. Sediments were sampled and processed according to a common protocol (details below).

The rationale for the sampling design is that we hypothesise that the River Danube is the primary pathway along which microplastics are transported across the environments studied. In common with recent studies, we assume that microplastic composition will be indicative of microplastic origin and we anticipate that microplastic distribution will decay with distance: first in sites sampled along the Lower Danube, and second in the Danube Delta with distance from the distributaries.

## Results

**Figure 2:**
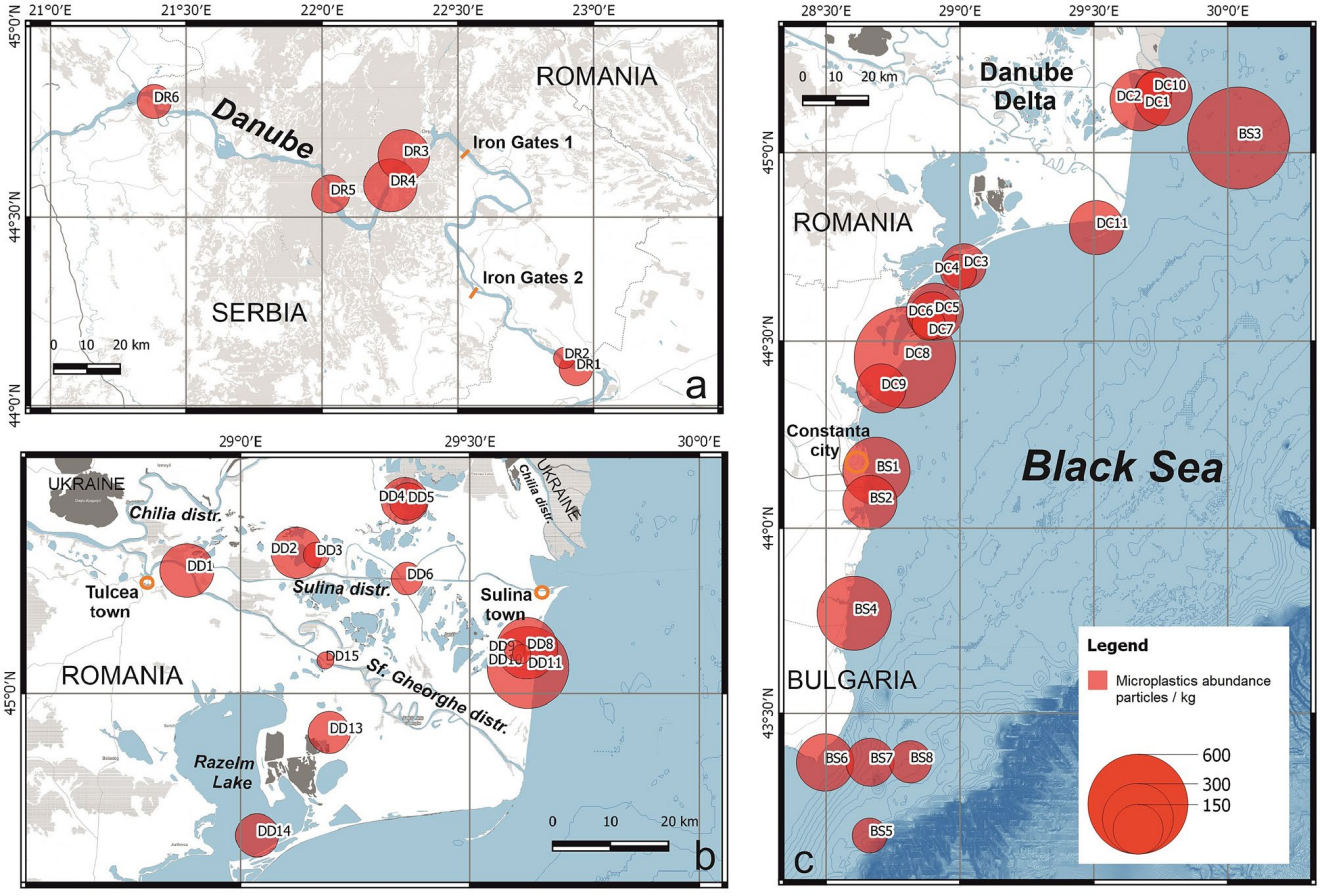
Microplastic particle abundance on: (**a**) the Danube River (Iron Gates area); (**b**) Danube Delta; and (**c**) Black Sea coast and inner shelf. The concentration is calculated for particles per 1 kg of sediment. (Figures created with the assistance of Cornel Pop, GeoEcoMar, using QGIS 3.4, background data: www.OpenStreetmap.org and https://land.copernicus.eu/).

Microplastic particles were found in all sediment samples (Fig. 2). The 38 samples yielded a total of 6047 particles kg^−1^ (mean: 159.2; st. dev.: 138.4). Microplastic concentrations across all sample locations varied over three orders of magnitude.

### Lower Danube

Low microplastic concentrations were found in two free-flowing reaches of the Danube River sampled upstream of Iron Gates I (< 87 particles kg^−1^), but increased by an order of magnitude immediately downstream, and subsequently decreased (by an order of magnitude) downstream of Iron Gates II dam (Fig. 2a).

### Danube Delta

In the Danube Delta, moderate microplastic concentrations (~ 165 particles kg^−1^) were observed at the bifurcation of the Sulina and Sf. Gheorghe Distributaries of the Danube below the city of Tulcea and at two sampling points on the Black Sea coast (Fig. 2b). Few microplastics (< 24 particles kg^−1^) were identified in the Sf. Gheorghe Distributary, and in Rosu Lake, but concentrations were higher (381 particles kg^−1^) in the channel to Rosu Lake. Microplastic concentrations were low in the Sulina Distributary of the Danube, upstream of the town of Sulina (< 59 particles kg^−1^). Microplastic concentrations were slightly higher in deltaic lake sediments sampled North of the Sulina Distributary (148 particles kg^−1^), but were low (< 40 particles kg^−1^) in the channel to Fortuna Lake.

### Black Sea: coast and marine sediments

Microplastic concentrations in beach sediments sampled along the Black Sea coast and in marine sands of the Bulgarian and Romanian internal shelf of the Black Sea varied over two orders of magnitude (Fig. 2c). Microplastic concentrations were highest on the Danube Delta coast at Chituc (~ 620 particles kg^−1^) with a mean coastal microplastic concentration elsewhere of 98 particles kg^−1^ (st. dev. 154). Marine microplastic concentrations were highest near the outlet of the Sf. Gheorghe Distributary (630 particles kg^−1^), and were lower in the Bulgarian Black Sea (mean 131; st. dev. 52).

### Microplastic composition

In river reaches along the Lower Danube and in the distributaries, channels and lakes of the Danube Delta, the microplastics sampled were mainly fibres (> 90%) with a small proportion of fragments and clumps (~ 3%). In contrast, the microplastic composition at sampling sites along the Black Sea coast were mainly flakes (80%), especially at Chituc where sediments were sampled within 150 m of a recent fire (of a building with plastic storage containers). The composition of microplastics in the Romanian Black Sea were mainly fibres (comparable to the composition of samples from the Danube River), while flakes predominated in the Bulgarian Black Sea with a small number of fibres and fragments.

## Discussion

At present, relatively little is known about the speciation, abundance and dynamics of microplastics along and across the freshwater–marine continuum, and we present here the first evidence of sedimentary microplastic concentration and composition along lower reaches of the River Danube, the Danube Delta and the inner shelf of the Black Sea. In contrast to a plethora of recent studies (e.g. Rhine and Main, Germany^26^, Yantgze Delta, China^27^), microplastic concentrations in sediments at our sample sites are an order of magnitude lower (such as for Qin River, 0–97 p/kg^−1^^28^). While microplastics were found at all sites sampled, some isolated sites in the Danube Delta had negligible concentrations and are still in relatively pristine condition. Microplastic concentrations were highest at the mouth of the Sulina Distributary on the Black Sea (630 particles kg^−1^). Microplastic abundance was also found to be highest in the estuary of the Qin River^28^. This is in accordance with the estimations of Lechner et al.^25^ for the Danube Delta. As the areas we sampled are sparsely populated, it is likely that the majority of microplastics sampled are largely derived from fluvial transport from distal sources, with the exception of the flakes observed at Chituc on the Danube Delta Black Sea coastline (620 particles kg^−1^) which were probably the product of aerial transport from a local fire. In contrast to other rivers (e.g. the Rhine, Germany^18^), the microplastics in sediments sampled along the Danube River–Danube Delta through to the Romanian Black Sea are predominantly fibres, which also pre-dominated in tidal flat sediments in Shanghai^12^. From this we infer that microplastic sources include poorly treated wastewater^29^, or inadequately treated sewage sludge^30^.

**Figure 3:**
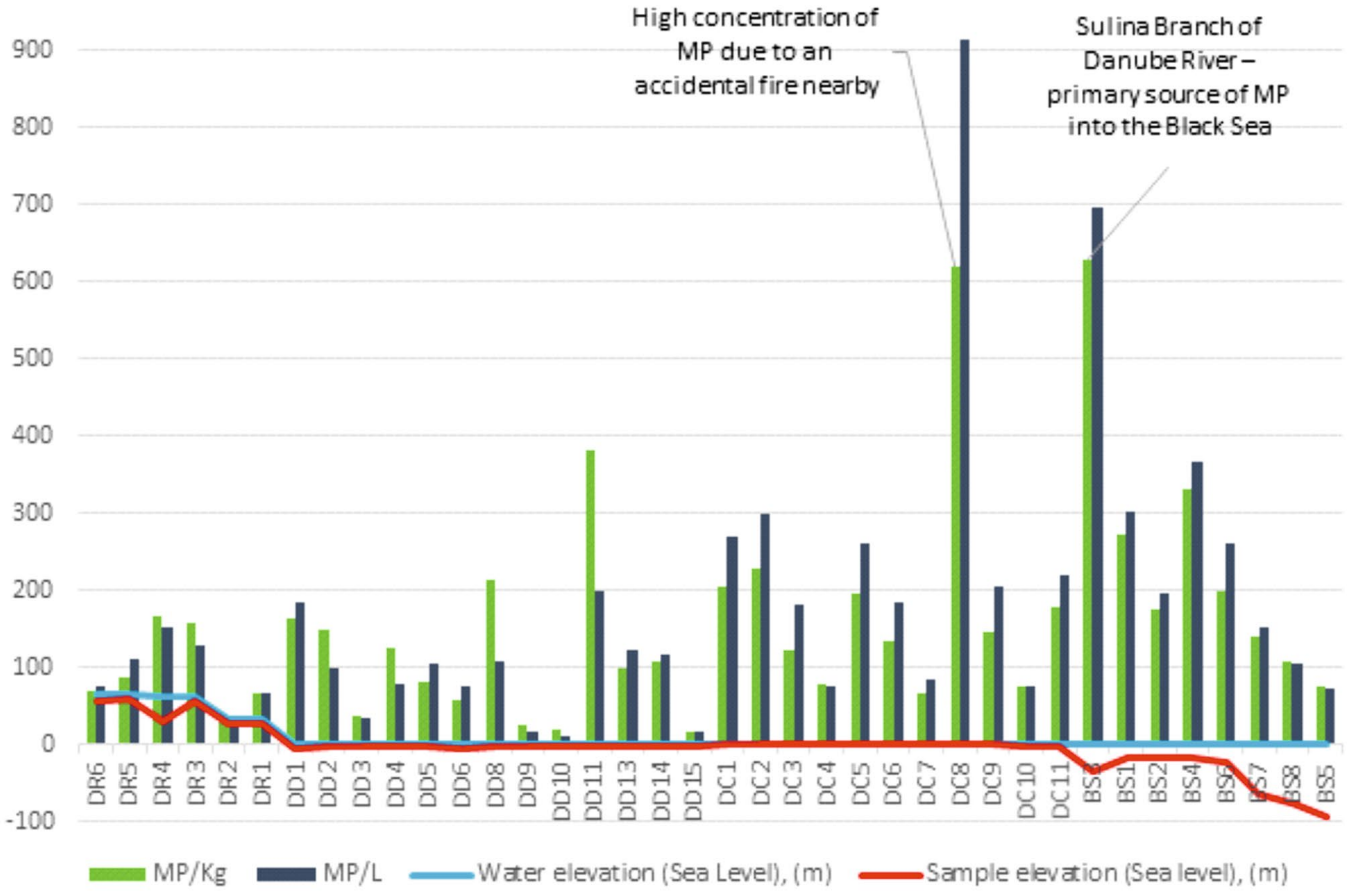
Microplastic particle abundance in the collected samples from the Danube River (left) to the Black Sea (right), showing the concentration in particles per 1 kg (green) and particles per 0.5 L (purple) of sediment. Also indicated are water level (blue) and sample elevation (red).

**Figure 4:**
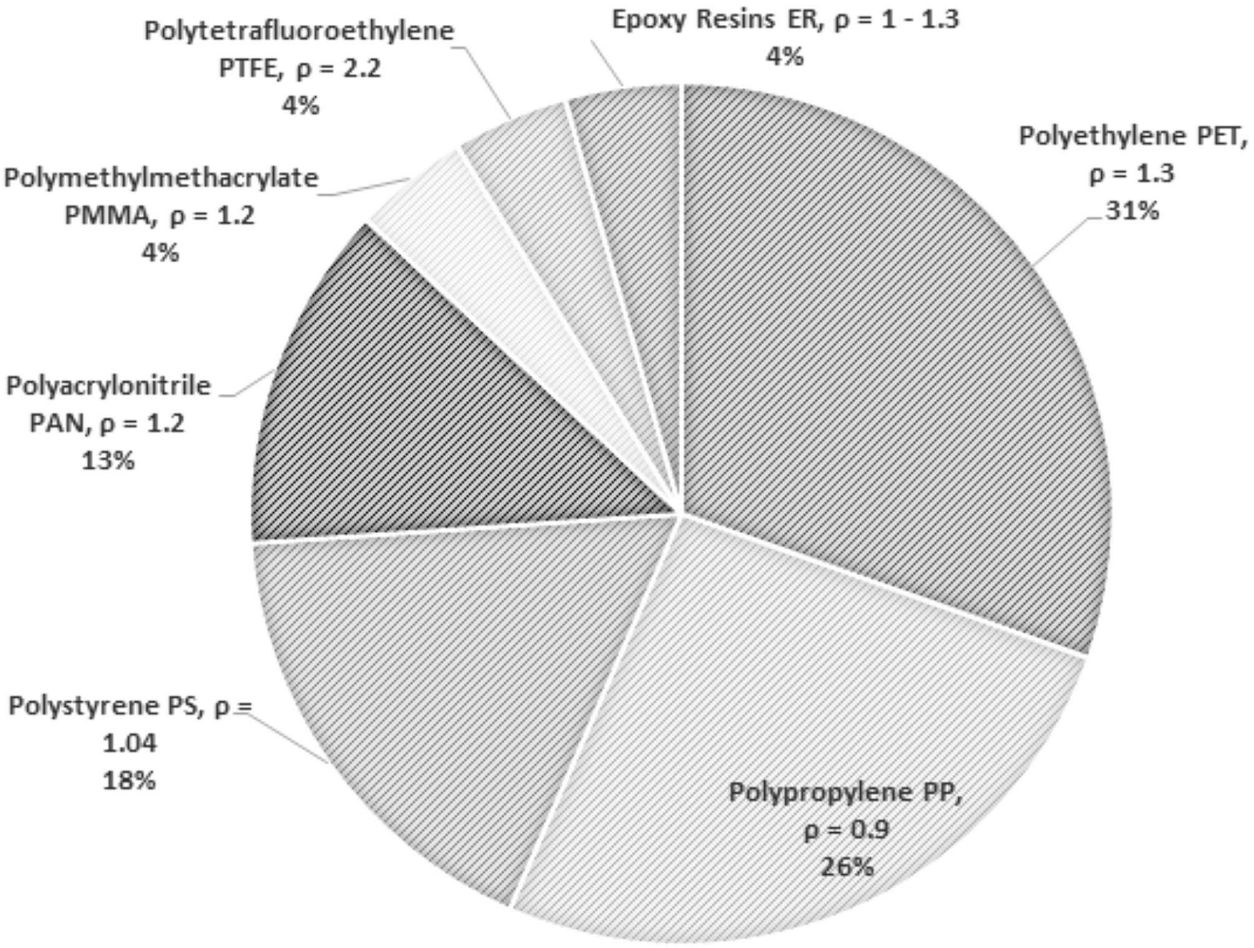
Polymers identified on Black Sea coast and shelf; density expressed in g/cm^3^.

It is difficult to reconcile the relatively low concentrations of microplastics in our sampling sites, particularly the negligible concentrations in lagoon lakes in the Danube Delta, with observations of high microplastic fluxes in the Danube River in Austria^25^. In the Austrian part of the Danube, however, only floating plastics have been investigated^25^. Moreover, nets with a mesh size of 500 µm were used and hence only larger particles are likely to have been sampled in this study. Our results suggest that high microplastic concentrations observed upstream have yet to translate into high concentrations in sediments downstream, and raises the question of where the missing microplastics may be accumulating (Fig. 3). In the Danube River Basin, upstream river regulation, hydropower development, and channelization have led to an estimated 60% reductions in sediment flux^31^ and the missing microplastics are most likely to be deposited in impounded reaches upstream, retained by barrages^31^ and in areas of active alluviation on the Danube floodplain, while polymers with a low specific gravity may be more widely distributed in the plume of Danube River waters in the Black Sea (Fig. 4). Other studies show that sediments are sinks where the plastic particles accumulate^12,32,33^. Moreover, the density might increase when transported in the water^34–36^ and plastic particles with a higher density might not be transported far along the river before they settle.

The results of the above-mentioned studies are comparable to our results. Sediments were taken with a corer or a grabber, samples purified and density-separated, visually identified and a selected number of particles analysed by means of µFTIR^26,37–39^ or pyrolysis for single particles (this study). Furthermore, particles with densities lower or equal than 1.2 g cm^−1^ (e.g. polypropylene, polystyrene, polyacrylonitrile and polymethylmethacrylate) represent ~ 65% of the microplastics we analysed, although the most common polymer was polyethylene. However, the low microplastic concentrations in the channels and lakes of the Danube Delta suggest limited flow exchange with the distributaries of the Danube, as there are few local sources of microplastics in the Danube Delta apart from scattered tourist lodges, and the nearest city, Tulcea, is 78 km W of the Black Sea.

**Figure 5:**
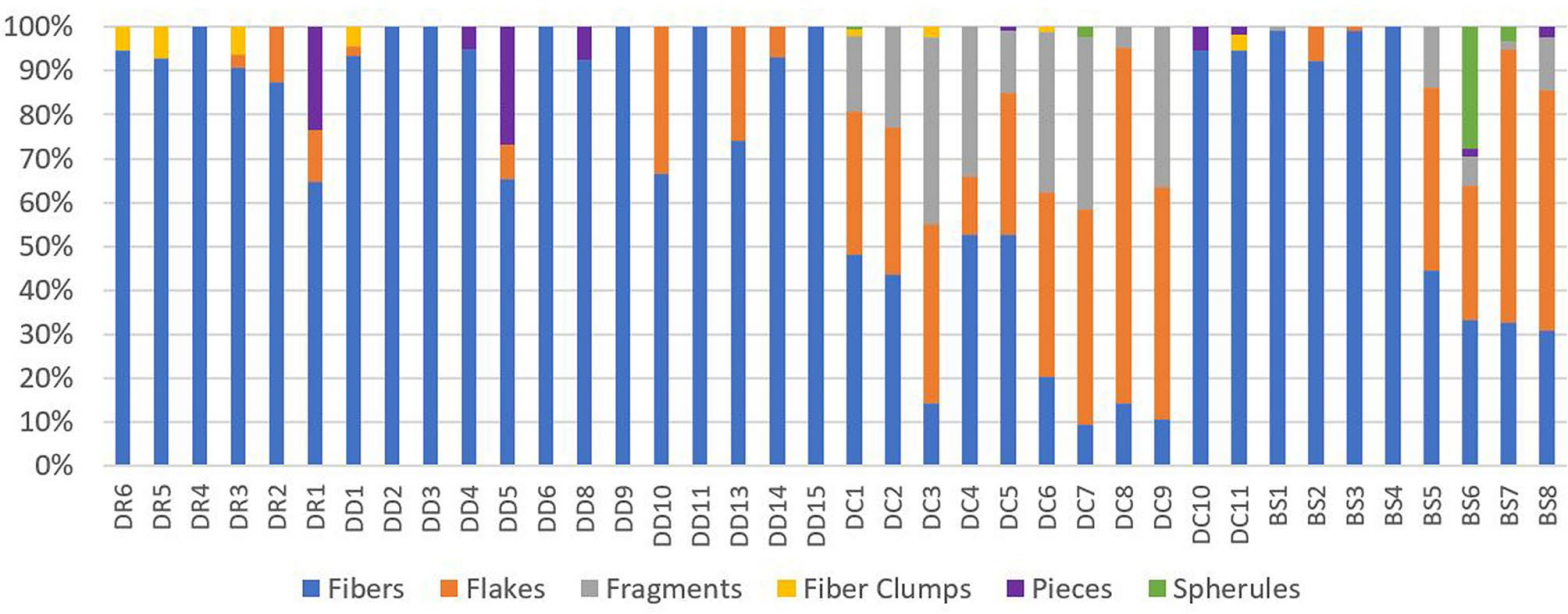
Observed microplastics morphology.

In the light of our results, further work is required to investigate the hydrodynamic controls on microplastic sedimentation, and to develop improved models of microplastic fluxes in coastal margins. These are necessary to determine the ultimate fate of riverine microplastic particles, and understand the differences in microplastic composition (fibres v. flakes) that we observed in sediments sampled between the Bulgarian and Romanian Black Sea shelf (Fig. 5). While the latter may reflect differences in the predominant microplastic sources, these patterns could arise due to variations in the buoyancy, density and shape of microplastics leading to preferential accumulation of certain microplastic types depending upon local hydrodynamic conditions.

## Methods

### Field methodology

Sites of active sedimentation along the Danube River in Romania, in the Danube Delta, along the NW Black Sea coast and on the inner shelf of the Black Sea were sampled during 5 field campaigns (March 2016: Danube River; April 2016: Danube Delta; June 2016: Romanian Black Sea; May 2017: Bulgarian Black Sea; May 2018: Black Sea coast) (Table S1) as part of a wider geo-ecological monitoring programme of the River Danube–Danube Delta–Black Sea coast and shelf (PN 16 45). Black Sea coast sediment samples (15 × 15 × 15 cm) were collected from the backshore using a shovel, immediately behind the active berm crest. All remaining samples were collected from a boat using an automated Van Veen type grab sampler for marine and riverine sites (39 × 31 cm; volume of 36 dm^3^ / 30 cm edge length), and a manual grab sampler for lacustrine sites in the Danube Delta (18 × 22 cm; volume of 8 dm^3^; 16 cm edge length) yielding sediment samples of ~ 1 dm^3^. Samples were stored in polypropylene bags for transport to the laboratories (NIRD GeoEcoMar, Constanța, Romania and Federal Institute of Hydrology, Koblenz, Germany).

### Rationale for sample location

Sampling locations were selected to represent major sedimentary environments (riverine, lacustrine, deltaic, coastal, continental, shelf) in the Danube River–Danube Delta–NW Black Sea coast and shelf.

Along the Danube River, sediments were sampled at 6 locations specified here in kilometres above Sulina (Table S1), where the Danube discharges into the Black Sea. Sample sites encompass three morphodynamic units: (1) the Lower Danube, which extends from km 965 (the Iron Gates I dam) to km 1075 (Austrian border). The upper reaches of the Lower Danube are free-flowing as the river crosses the Carpathian Mountains before progressing to: (2) a lacustrine environment in the barrage lake upstream Iron Gates I. The lacustrine environment extends downstream from km 965 to Iron Gates II at km 863. (3) Below km 863 the Lower Danube comprises a multi-channel river system where the river is largely free to migrate laterally across the floodplain. There is little bank protection, although the channel is locally constrained in the vicinity of agricultural polders on the floodplain to the North. Below km 87, the Danube flows through the Danube Delta along the three distributaries: Chilia, Sulina and Sf. Gheorghe (Fig. 1a; Table S1).

Sampling locations in the Danube Delta included channels and lakes in transitional environments spanning a salinity gradient. Sites were selected to include a hierarchy of locations situated at progressively increasing distances from the three distributaries : 1st order lakes are connected to a distributary via a single channel; 2nd order lakes are connected to a distributary via a channel, an intermediate lake and a second channel; while 3rd order lakes are connected to a distributary via two intermediate lakes and three channels. Sediments were sampled at 10 points in the Danube Delta (Fig. 1b, Table S2), with individual locations specified here by their distance (by channel) from a distributary. Two replicate samples were collected from Fortuna (1st order lake) and Matita (2nd order lake) Lakes and, for each lake, from the channel between the Chilia and Sulina distributaries of the Danube. Two replicate samples were collected from the mouth of the channel (Tataru Canal) connecting Rosu Lake (3rd order lake) with the Sulina Distributary.

Along the Black Sea coast, sampling locations were selected to investigate the variation in microplastic concentration with distance from the mouths of the Danube distributaries, and determine whether microplastic concentrations were affected by flows from Edighiol lagoon (Table S3). Sediments were sampled from: (1) a sand shoal on the margin of the Sulina Distributary; (2) an area of the beach backshore with active accretion of sediment from the River Danube^40^; (3) an area of north to south longshore sediment transport, 66 km S of the Sf. Gheorghe Distributary and an area of local secondary circulation 60 km S of the mouth of the Sf. Gheorghe Distributary. Points north and south of the Edighiol Inlet of the Sinoie Lagoon were sampled to investigate microplastic concentrations in areas potentially influenced by water fluxes from the RazelmSinoie Lagoon System. Beach sediments were also sampled in the Southern Danube Delta: Chituc is characterised by an active longshore sediment transfer, and Cape Midia, where sediment fluxes are impeded by harbour protection (jetties).

Sediments along the north-western inner shelf of the Black Sea were sampled to investigate the extent of microplastic fluxes from the River Danube: at the mouth of the Sulina distributary channel; adjacent to the southern offshore boundary of the Danube Delta Biosphere Reserve (including a point close to the city of Constanța). Sediments were also sampled from the inner shelf of the Black Sea; in Bulgarian and Romanian territorial waters (Table S4). Several sediment samples were collected from the north-western Black Sea inner shelf, in order to understand riverine inputs of microplastic particles and their distribution. One sample was in the close vicinity of the Danube mouth of Sulina (NR), with two more at the southern offshore boundary of the Danube Delta Biosphere Reserve (one of them South of the major city and harbour of Constanța). Two further sediment samples were collected and analysed from the western part of the Black Sea shelf, in the territorial waters of the Republic of Bulgaria.

### Laboratory processing

#### Sample preparation

All the laboratory analyses detailed here were undertaken by IP (the first author). IP was trained to the state-of-the-art in MP analysis by the co-authors in BfG and therefore the same methodological concept was used for all samples. Of the samples collected, 25 were processed at GeoEcoMar’s geochemistry laboratory, and 13 (samples from the Black Sea Coast and the Bulgarian Shelf) were processed at the German Federal Institute of Hydrology. Standardised sub-samples of ~ 500 ml were taken and oven-dried at 60 °C for 24 h and sediment composition noted (detailed in Tables S1-4).

At GeoEcoMar, sample processing comprised: sieving through a 5 mm sieve (the upper limit for microplastics^2^; Arthur et al*.,* 2009). A volume of 250 to 500 ml of sediment was mixed with saline solution (NaCl reaching a maximum density of 1.2 g/cm^3^) using a ratio of 1:4^41^. The solution was mixed for 15 min and allowed to settle for a further 15 min, until the mixture was clear, prior to extracting the mixture of saline solution and floating material. The extract was then vacuum-filtered through 1 µm polycarbonate filters (47 mm Ø)^42^ and deposited in Petri dishes before treatment with 30% H_2_O_2_ for 7 days^43^. In some cases, organic matter had not completely dissolved but this was sufficient to provide clear observations using a stereo microscope.

A major discrepancy between the analytical processes for sample preparation performed at GeoEcoMar and German Federal Institute of Hydrology laboratories represents the usage of different density separation liquids. Therefore, using sodium chloride (1.2 g/cm^−3^), several polymers [e.g. polyethene terephthalate (PET, 1.38–1.41 g/cm^−3^), polyvinyl chloride (PVC, 1.38–1.41 g/cm^−3^), polytetrafluoroethylene (PTFE, 2.10–2.30 g/cm^−3^)] could be absent from the samples processed at GeoEcoMar. Compositional details of the samples introduced in NaCl (samples DR1-6, DD1-15 and BS1-4) for density separation, indeed reveal higher abundances in fiber morphological type, although no polymer differences were observed between these samples and those separated with K(HCOO) (potassium formate; in Germany).

Sample processing in Germany comprised first, oven drying (as above) as sieving was not required given the sedimentary composition. Second, sample volumes (and the solution quantities for digestion) were reduced using an electrostatic separation method^41,44^. The device comprised a conveyer with a programmable vibration speed which allowed sediment to ‘flow’ to an earthed drum. Particles were charged electrostatically at 20 kV whilst under an electric field to separate particles with differing electrostatic properties, enabling plastics and nonconductive particles to be discarded. Several runs were required: first at a conveyor vibration speed of 900 vibes/min. (15% of vibration capacity), drum speed of 66.5 rpm (50% of capacity) and a voltage of 20 kV; second run, only for sediments previously categorized as magnetic, at a vibration speed of 600 vibes/min, drum speed 66.5 rmp and voltage 20 kV; third the nonmagnetic fractions from runs 1 and 2 were processed using the same parameters as the second run (vibration speed of 600 vibes/min, drum speed of 66.5 rpm and a voltage of 20 kV). Electrostatic processing enabled sample volumes to be reduced by up to 80% (to a final volume of ~ 100 ml)^44^.

Sample digestion used equal volumes of KOH (10 M) and H_2_O_2_ (35%) with a total volume twice that of the sample (i.e. for a sediment sample of 100 ml, 100 ml of KOH and 100 ml of H_2_O_2_ were added). The mixture was agitated for between 5 and 7 days before neutralising: first by adding ultra-filtrated distilled water until the volume had doubled; and second, by adding HCOOH at a ratio of 0.385:1 (i.e. for 10 ml of H_2_O_2_, 3.85 ml of HCOOH was added; )^45^. The neutralised mixture was then added to a separating funnel for density separation using potassium formate (c. 365 g of (K(HCOO))/CHKO_2_ for 100 ml of mixture)^46^. The solution was filtered using 1 µm glass fibre membranes to capture floating microplastic particles from the supernatant.

For sediment sample analysis we used volume units and, subsequently, weighed each sample after drying, before electrostatic separation or digestion in order to derive microplastic abundance in mass units.

#### Visual identification

The membranes were observed using a digital microscope (Keyence—VHX-2000) equipped with a 200 × lens. All particles ranging between 20 µm and 5 mm were analysed to quantify their morphology, dimensions and colour. Particle abundance was based on filtered water and microplastic particles per kg or 0.5L. 50 particles were selected for spectrometric analysis from all processed samples.

#### Pyrolysis GC–MS tests

A sub-sample of 50 particles were analysed using a pyr-GC–MS: particles were placed in pyrolysis cups and flashed at 600 °C using a Multi-Shot Pyrolyzer EGA/PY-3030D (Frontier Laboratories, Saikon, Japan) and an Auto-Shot Sampler AS-1020E (Frontier Laboratories, Saikon, Japan). Pyrolysis products were separated by an Agilent7890B GC (Agilent Technologies, Santa Clara, USA) equipped with an Ultra ALLOY UA-5(MS/HT) metal capillary separation column (Frontier Laboratories, Saikon, Japan) with dimensions 30 m length, 0.25 mm ID and 0.25 µm film thicknesses. The split ratio was 1:50 for particles and 20:1 for fibres. Chromatographic separation was performed using the following temperature programme: hold at 40 °C for 2 min, increase at 20 °C min^−1^ to 320 °C and hold for 13 min. Particles were detected by high resolution MS using a 7200 Q-ToF (Agilent Technologies, Santa Clara, US) to characterize particle polymers using comparison data from the library F-Search 3.4 (Frontier Laboratories, Saikon, Japan).

#### Quality assurance

Laboratory contamination was minimised through the use of glass and stainless-steel products in the laboratory. The work space was cleaned immediately prior to each process, and cotton laboratory coats were worn. During sample preparation, four blank samples were included, using the same quantities of H_2_O_2_, K(HCOO), HCOOH and distilled water, digested and filtered in the same way as the other samples. Airborne fibre contamination during laboratory analysis and microscopic identification was detected in the blank samples with a mean concentration of 85. After quantification the necessary corrections were applied to each filter with supplementary corrections for each morphological type.

## Supplementary Information

Supplementary Information.

Supplementary Information.
