# Supplementary material for: Sedimentary microplastic concentrations from the Romanian Danube River to the Black Sea
Source: Sci Rep. 2021 Jan 21;11:2000. doi: 10.1038/s41598-021-81724-4 (PMC7820245; doi:10.1038/s41598-021-81724-4)
Supplement: Supplementary file 1 — Supplementary Information. [file 41598_2021_81724_MOESM1_ESM.docx]

Sedimentary microplastic concentrations from the Romanian Danube River to the Black Sea

Iulian POJAR, Adrian STĂNICĂ, Friederike STOCK, Christian KOCHLEUS,

Michael SCHULTZ & Chris BRADLEY

Supplementary Materials

Table S1. Locations and characteristics of the Danube River sampling points: DR1: DR6

| Sample no. | Loc. description; km. upstream Sulina; local characteristics | Distance from the left bank; bankfull width of the Danube River at sample point | Sediment type | Water Depth (m) |
| --- | --- | --- | --- | --- |
| DR1 | Downstream of II^nd^ dam; km 820; multi-channel system, Lower Danube | 790m; 835m | Sand | 3.9 |
| DR2 | Downstream II^nd^ dam; km 824; multi-channel, Lower Danube | 35m; 1265m | Sandy silt | 3.95 |
| DR3 | Upper reaches of the I^st^ dam lake; km 965; lacustrine environment, Middle Danube | 170m; 1240m | Silt | 5.14 |
| DR4 | Upstream I^st^ dam; km 974; fast – stream river flow, Middle Danube | 671m; 758m | Silt | 32.87 |
| DR5 | Fastest river flow point; km 1005; free – flowing river section, Middle Danube | 75m; 515m | Sand | 6.25 |
| DR6 | km 1072; free-flowing river section, Middle Danube | 735m; 885m | Sand | 9.13 |

Table S2. Locations of sites sampled in the Danube Delta

| Sample no. | Sample location and  system characterisation | Sediment type | Order / Rank | Water depth (m) |
| --- | --- | --- | --- | --- |
| DD1 | Ceatal Sf. Gheorghe; fluvial; km. 61 | Sandy mud | - | 4.5 |
| DD2 | Fortuna Lake; deltaic | Mud | 1 | 1.1 |
| DD3 | Fortuna Lake channel; deltaic | Mud | 1 | 0.5 |
| DD4 | Matita Lake channel; deltaic | Mud | 2 | 0.5 |
| DD5 | Matita Lake; deltaic | Mud | 2 | 2.6 |
| DD6 | Sulina distributary; fluvial; km 57.5 | Mud | - | 0.5 |
| DC10 | Sulina distributary; coastal | Mud | - | 1.3 |
| DD8 | Rosu Lake channel; deltaic | Mud | 3 | 0.5 |
| DD9 | Rosu Lake; deltaic | Mud | 3 | 0.5 |
| DD10 | Rosu Lake; deltaic | Mud | 3 | 0.5 |
| DD11 | Rosu Lake channel; deltaic | Mud | 3 | 0.5 |
| DC11 | Sahalin Coastal Lagoon; coastal | Sand | - | 0.5 |
| DD13 | Razelm Lake channel; deltaic | Sand | 4 | 0.5 |
| DD14 | Razelm Lake; deltaic | Sandy mud | 4 | 0.5 |
| DD15 | Sf. Gheorghe distributary; fluvial; km 0.4 | Sand | - | 0.5 |

Table S3. Details of sampling points on the Black Sea coast

| **Sample** | **Sample location** | **Sediment type** |
| --- | --- | --- |
| DC1 | 6.5 km SW of the mouth of the Sulina distributary | Sand |
| DC2 | 1.7 km SE of Sulina Town | Sand |
| DC3 | 1.5 km NE of Gura Portitei | Sand |
| DC4 | 1.0 km SW of Gura Portitei | Sand |
| DC5 | 15.2 km below the canal linking Sinoe Lake to the Black Sea | Sand |
| DC6 | near Periboina (canal linking Sinoe Lake to the Black Sea) | Sand |
| DC7 | 3.3 km below the canal linking Sinoe Lake to the Black Sea | Sand |
| DC8 | 26.5 km below the canal linking Sinoe Lake to the Black Sea | Sand |
| DC9 | 10 km below the canal linking Sinoe Lake to the Black Sea | Sand |
| DC10 | Musura Bay - influenced by the Sulina distributary; depth 1.3m | Sand |
| DC11 | Sahalin Bay - influenced by the Sf. Gheorghe distributary; depth 2.5m | Sand |

Table S4. Sample locations on the Black Sea Inner Shelf

| **Sampled area** | **Sample** | **Distance from the closest point from shore** | **Sediment type** | **Depth (m)** |
| --- | --- | --- | --- | --- |
| Black Sea Inner Shelf (Romania) | BS1 | 1.3 km E of Constanta City | Sand | 16.5 |
|  | BS2 | 1.8 km E of Eforie Nord | Sand | 16.0 |
|  | BS3 | 24.5 km E of Sulina Arm | Sand | 34.7 |
|  | BS4 | 2.1 km E of the coast between 2 Mai and Vama | Sand | 16.5 |
| Black Sea Inner Shelf (Bulgaria) | BS5 | 27.2 km SE of Kaliakra Cape | Mud | 94.4 |
|  | BS6 | 2.4 km E of Kaliakra Cape | Sand | 23.2 |
|  | BS7 | 16 km E of Kaliakra Cape | Muddy sand | 63.7 |
|  | BS8 | 28,2 km E of Kaliakra Cape | Mud | 74.8 |

Table S5. GPS coordinates of the sampled locations. Coordinates expressed in degrees and decimals.

| **Sample No.** | **Latitude (degrees, decimals)** | **Longitude (degrees, decimals)** |
| --- | --- | --- |
| DR1 | 22.93683333 | 44.09694444 |
| DR2 | 22.89391667 | 44.12544444 |
| DR3 | 22.30152778 | 44.66069444 |
| DR4 | 22.25327778 | 44.58283333 |
| DR5 | 22.033 | 44.55869444 |
| DR6 | 21.3825 | 44.80255556 |
| DD1 | 28.88425 | 45.18838889 |
| DD2 | 29.12322222 | 45.21563889 |
| DD3 | 29.16633333 | 45.21247222 |
| DD4 | 29.35838889 | 45.29536111 |
| DD5 | 29.36577778 | 45.29383333 |
| DD6 | 29.36377778 | 45.17605556 |
| DD8 | 29.62558333 | 45.06847222 |
| DD9 | 29.59813889 | 45.06536111 |
| DD10 | 29.61405 | 45.06008333 |
| DD11 | 29.62788889 | 45.03941667 |
| DD13 | 29.19402778 | 44.93880556 |
| DD14 | 29.03716667 | 44.78019444 |
| DD15 | 29.18538889 | 45.04988889 |
| DC1 | 29.76071667 | 45.14743333 |
| DC2 | 29.67702325 | 45.13740526 |
| DC3 | 29.01522319 | 44.69907263 |
| DC4 | 28.99653854 | 44.68242685 |
| DC5 | 28.90711722 | 44.57748838 |
| DC6 | 28.89983488 | 44.56830954 |
| DC7 | 28.88400625 | 44.54498335 |
| DC8 | 28.79611664 | 44.45513684 |
| DC9 | 28.70758818 | 44.37234734 |
| DC10 | 29.72641667 | 45.16586111 |
| DC11 | 29.51052778 | 44.80102778 |
| BS1 | 28.68985 | 44.15271667 |
| BS2 | 28.66405 | 44.06888333 |
| BS3 | 30.04158333 | 45.03768333 |
| BS4 | 28.60655 | 43.76951667 |
| BS5 | 28.66496667 | 43.16736667 |
| BS6 | 28.49783333 | 43.36581667 |
| BS7 | 28.6661 | 43.36625 |
| BS8 | 28.81596667 | 43.36733333 |
